# Did limits on payments for tobacco placements in US movies affect how movies are made?

**DOI:** 10.1136/tobaccocontrol-2015-052400

**Published:** 2016-01-28

**Authors:** Matthis Morgenstern, Mike Stoolmiller, Elaina Bergamini, James D Sargent

**Affiliations:** 1 Norris Cotton Cancer Center, Geisel School of Medicine at Dartmouth, Lebanon, New Hampshire, USA; 2 Institute for Therapy and Health Research, Kiel, Germany; 3 Department of Pediatrics, College of Human Medicine, Michigan State University, Ann Arbor, Michigan, USA; 4 Department of Pediatrics, Geisel School of Medicine at Dartmouth, Lebanon, New Hampshire, USA

**Keywords:** Media, Tobacco industry, Public policy

## Abstract

**Objective:**

To compare how smoking was depicted in Hollywood movies before and after an intervention limiting paid product placement for cigarette brands.

**Design:**

Correlational analysis.

**Setting/Participants:**

Top box office hits released in the USA primarily between 1988 and 2011 (n=2134).

**Intervention:**

The Master Settlement Agreement (MSA), implemented in 1998.

**Main outcome measures:**

This study analyses trends for whether or not movies depicted smoking, and among movies with smoking, counts for character smoking scenes and average smoking scene duration.

**Results:**

There was no detectable trend for any measure prior to the MSA. In 1999, 79% of movies contained smoking, and movies with smoking contained 8 scenes of character smoking, with the average duration of a character smoking scene being 81 s. After the MSA, there were significant negative post-MSA changes (p<0.05) for linear trends in proportion of movies with any smoking (which declined to 41% by 2011) and, in movies with smoking, counts of character smoking scenes (which declined to 4 by 2011). Between 1999 and 2000, there was an immediate and dramatic drop in average length of a character smoking scene, which decreased to 19 s, and remained there for the duration of the study. The probability that the drop of −62.5 (95% CI −55.1 to −70.0) seconds was due to chance was p<10^−16^.

**Conclusions:**

This study's correlational data suggest that restricting payments for tobacco product placement coincided with profound changes in the duration of smoking depictions in movies.

## Introduction

A 2012 Surgeon General's Report contains the following causal statement about movies and youth tobacco use in chapter 5: “The evidence is sufficient to conclude that there is a causal relationship between depictions of smoking in the movies and the initiation of smoking among young people”.^1^ This evidence has resulted in scrutiny of the movie industry, with attention to how payments affect smoking in movies.^2^ If payments are found to dramatically alter the depiction of tobacco in film, this fact would undermine the idea that smoking in movies is primarily artistic in nature and speak for policies to restrict such practices, given the US Surgeon General's causal statement.^3^ ^4^

In the 1998 Master Settlement Agreement (MSA), the 46 State Attorneys General and the major tobacco companies agreed to end tobacco company payment for paid product placements of their brands in film or television produced in the USA,^5^ which was associated with an exponential decline in tobacco brand appearances in Hollywood movies in the years after it was signed.^6^ ^7^ However, non-branded depiction of smoking is not necessarily commercial in nature. To the extent that non-branded screen smoking is purely artistic, one would not expect to find major changes in these types of depictions to be associated with the MSA.

This study examines movie screen tobacco use in the context of the timing of the MSA. The overarching hypothesis is that paid product placement affected brand placements and may also have affected how character smoking was depicted generally. To test the hypothesis, we assessed whether the signing of the MSA was associated with three metrics: the proportion of movies with smoking, and among movies with any smoking, the number of scenes in which characters smoked, and the average length of a smoking scene. Movies take 1–2 years to produce, so we hypothesised a 1 year lag for the MSA to impact how smoking was depicted.^8^

## Methods

### Sample selection

The Dartmouth Media Laboratory content coded movies for the years 1988–2011; movies with the highest US box office gross revenues were selected. Prior to 1996, the content coding was restricted to the top 25 box office hits, and afterwards it included the top 100 box office hits, plus an additional 10–15 movies each year. The same two trained coders were employed through most of the time period; they viewed theatre versions of movies on videotape or DVD. They counted the number of scenes in which any major or minor character smoked and timed the duration of each smoking scene in minutes. A scene represented a continuous passage of time demarcated by an abrupt shift in time or location. ‘Character use’ included tobacco use or handling by movie characters; characters were identified by the coders after a first look at the movie as any actor whose presence or actions had something to do with the plot. Character use did not include smoking depictions by extras that occurred in the background in, for example, a bar scene.

We assessed first whether there was any depiction of smoking in the movie. In any movie with smoking, we then counted scenes of smoking and timed smoking depiction by characters in those scenes. ‘Character use’ included tobacco use or handling by major or minor characters. Major characters are those who played leading roles and who are essential to the development of the plot. Minor characters are those who played an important role in the movie, but are not central characters in the story. Timing involved screen time for all character depictions in the entire movie. For example, if a character smoked a cigarette during one scene that lasted several minutes, and the cigarette appeared on-screen twice for 3 s each time, this would count as one character smoking scene and tobacco exposure time would be 6 s. If a scene contained 30 s of smoking by two characters whose smoking overlapped by 10 s, this would be two character smoking scenes and the total smoking exposure time was recorded as 50 s. For each movie, the average tobacco duration for a character smoking scene was determined by dividing total tobacco time by the overall number of scenes.

To evaluate inter-rater reliability, a random sample of 10% of the movies was examined by both coders. The correlation of counts between the coders was 0.99 for major and minor character use and 0.92 for background smoking. Cohen's κ was used to assess inter-rater reliability for whether the timer was on or off for consecutive 5 s blocks of tobacco time for each movie. Average κ among movies was 0.97 for tobacco time; however, there was a range in κ values among movies, with movies with less smoking having lower average κs. The results from these content coding activities have been previously published multiple times since 2001.

### Statistical analysis

The focus of the analysis was abrupt change in average level or trend after onset of the MSA for (1) the proportion of movies with smoking, and (2) the number of scenes in which characters smoked (in movies with any smoking), and (3) the average length of a character smoking scene (in movies with character smoking). The specific time point hypothesised for change was after 1999, about 1 year after MSA implementation. A 1 year lag was chosen to represent the average time from production to movie release. Trends before and after 1999 were fitted non-parametrically and independently of each other using generalised additive models (GAM), an approach that is statistically equivalent to analyses for regression discontinuity designs, which are well developed.^9^ The GAM for presence of movie smoking used a logit link and binomial distribution; the GAM for character smoking scenes in movies with any smoking used a log link and overdispersed Poisson distribution, and the GAM for average character smoking scene length used a log link and γ distribution. GAM was implemented using the MGCV package in R.^10^

## Results

### Description of the movie sample

The 2134 movies were primarily produced or distributed by the major Hollywood studios (there were only 4 foreign films). The clear majority of successful movies of these years had PG-13 or R ratings. On average, 69% of movies had smoking; in movies with any smoking, there were 6.8 character smoking scenes and the average length of a character smoking scene was 43.3 s (table 1).

**Table 1:** Selected movie characteristics (1988–2011)

	All years	Pre 2000	Post 1999
Total, n (%)	2134 (100%)	680 (32%)	1454 (68%)
Motion picture rating, n (%)			
General	91 (4.3%)	27 (4.0%)	27 (4.4%)
PG	380 (17.8%)	129 (19.0%)	129 (17.3%)
PG-13	881 (41.3%)	209 (30.7%)	209 (46.2%)
Restricted	782 (36.6%)	315 (46.3%)	315 (32.1%)
Smoking in movies			
Movies with any smoking, n (%)	1465 (69%)	571 (84%)	894 (61%)
Scenes with character smoking*, mean (SD)	6.8 (SD 8.8)	7.9 (SD 10.3)	6.1 (SD 7.7)
Character smoking average scene length†, mean (SD)	43.3 (SD 44.5)	80.0 (SD 48.1)	18.9 (SD 16.4)

*Considers only movies with smoking.

†In seconds and considers only movies with at least 1 character smoking scene (n=1267).

### Trend analysis

**Figure 1:**
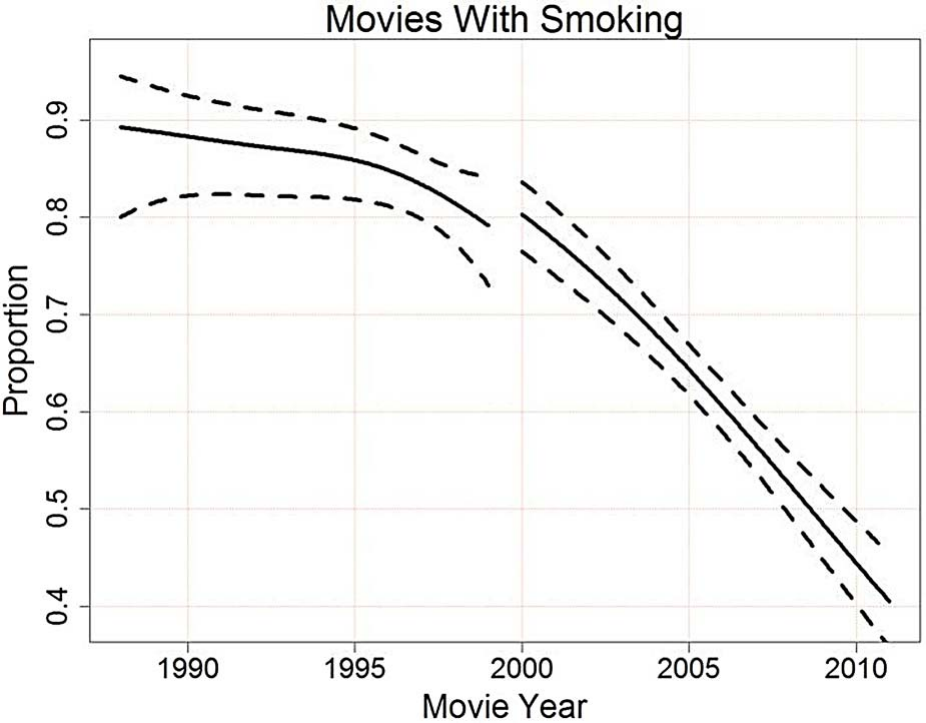
Fitted generalised additive model (GAM) for any smoking in a movie using logistic link and binomial distribution. Trends pre and post 1999 fit independently of each other. Dashed lines represent pointwise 95% CIs.

Fitted GAM plots for the proportion of movies with smoking are shown in figure 1. The curve prior to 1999 shows a slight downward trend that was not statistically significant. In 1999, 79% of movies contained smoking. After the MSA in 2000, the proportion of movies with any smoking was 80%, not significantly different from 1999 and the proportion trended downward, such that they had declined to 41% by 2011. The linear trend for the annual decrease in per cent of movies with smoking from 2000 to 2011 was −3.6% (95% CI −3.0% to −4.2%), and the p value comparing slope before and after 1999 was 0.028.

**Figure 2:**
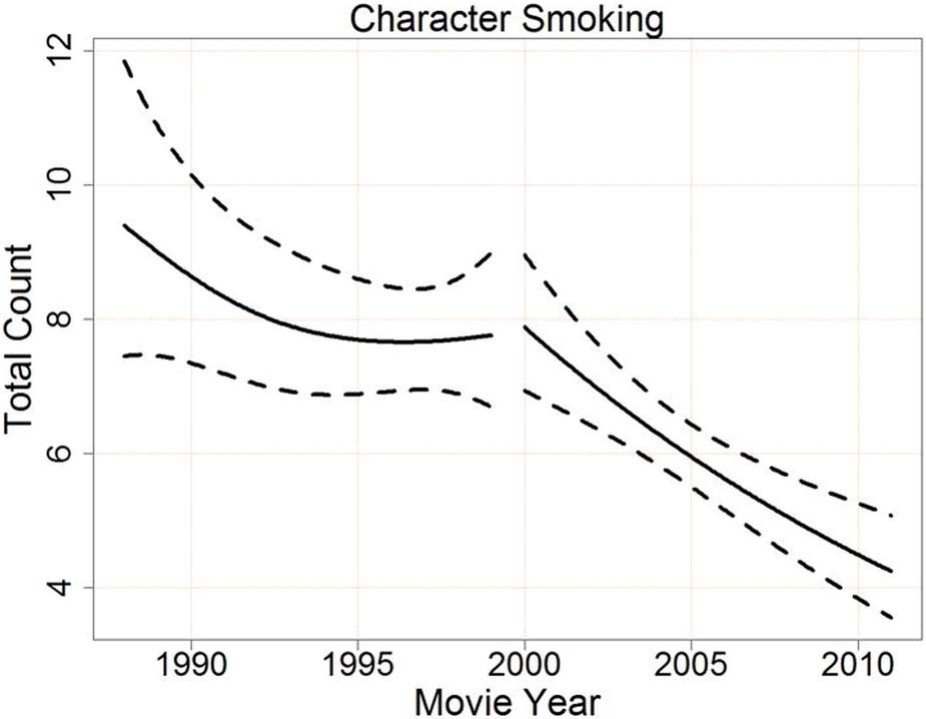
Fitted generalised additive model (GAM) for total number of character smoking scenes in a movie with any smoking using log link and overdispersed Poisson distribution. Trends pre and post 1999 fit independently of each other. Dashed lines represent pointwise 95% CIs.

The number of scenes with character smoking (among movies with smoking) are shown in figure 2. The curve prior to 1999 shows a slight downward trend that was not statistically significant. In 1999, movies with smoking contained 7.8 scenes of character smoking. After the MSA in 2000, movies with smoking contained 7.9 scenes of character smoking, not significantly different from 1999, and counts of characters smoking trended downward, such that they declined to 4.2 by 2011. The linear trend for annual decrease in counts of character smoking from 2000 to 2011 was −0.33 (95% CI −0.19 to −0.47), and the p value comparing slope before and after 1999 was 0.032.

**Figure 3:**
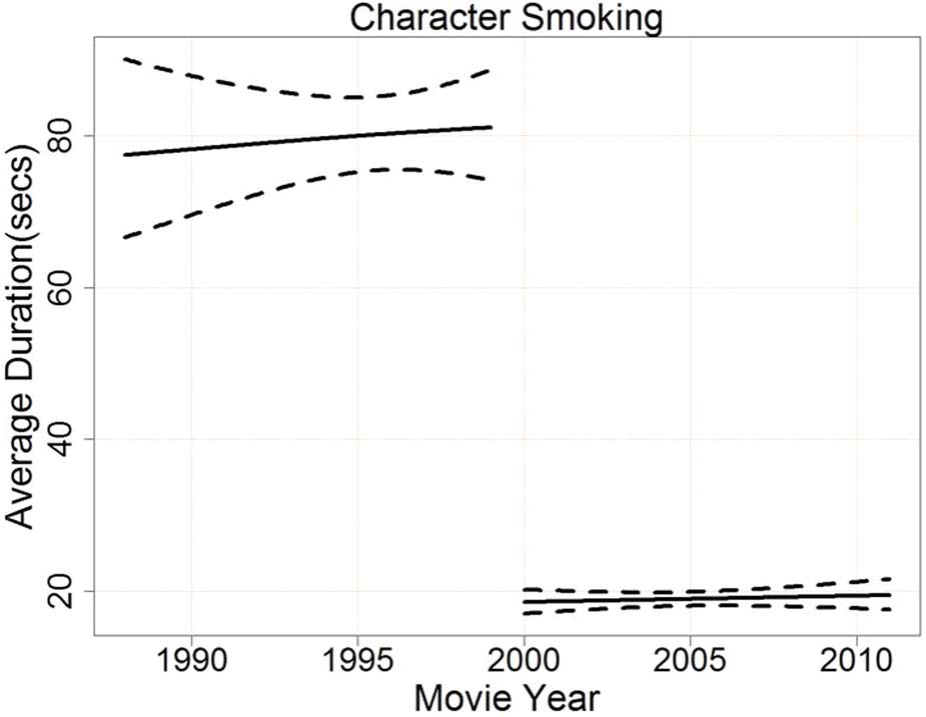
Fitted generalised additive model (GAM) for average duration of character smoking scenes in movies with character smoking using log link and γ distribution. Trends pre and post 1999 fit independently of each other. Dashed lines represent pointwise 95% CIs.

Figure 3 displays fitted GAM plots for the average duration of a character smoking scene. Different from the other two metrics, there was no significant trend for curves before or after 1999. However, between 1999 and 2000, there was an immediate and dramatic drop in the average length of a character smoking scene, which decreased by 77% (from 81 to 19 s) and remained there for the duration of the study. The total drop in character smoking scene length was −62.5 s (95% CI −55.1 to −70.0); the probability that this discontinuity was due to chance was p<10^−16^.

A sensitivity analysis tested if the results with respect to abrupt changes in mean level pre-MSA and post-MSA were influenced by Motion Picture Association of America (MPAA) rating. An analysis that stratified by the three categories of G or PG, PG-13 and R revealed no significant differences in mean level change pre-MSA and post-MSA across the three categories for any outcome. Detailed results are available on request.

## Discussion

This study offers strong circumstantial evidence that an externally enforced agreement prohibiting payments for tobacco brand placements was followed by reductions in a very specific aspect of movie making—the time devoted to character smoking depictions. The MSA was followed by an abrupt decrease in the average length of a smoking scene, a decline that occurred between 1999 and 2000, roughly when the MSA should have affected US movies coming to market. This decline was not preceded or followed by any significant trend in this metric that could form an alternative explanation. Given the timing and the size of this change, along with the previously documented abrupt decline in tobacco brand placements,^6^ it is tempting, though speculative, to conclude that the reduced smoking screen time occurred because there was no longer a financial incentive to work a cigarette brand into scenes where characters smoked.

Regulation of movie smoking has been controversial because movies are a mixed media, with artistic and commercial elements. Members of the public health community emphasise the role that the tobacco industry has played historically in ensuring that smoking and brand depiction was integrated into movies.^11^ Critics of restricting smoking in movies have suggested that “an equally plausible explanation for smoking in movies is that many movie directors are attuned to the richly signifying semiotics of smoking and often judge that characters should smoke to convey particular associations”.^12^ On the contrary, results from this study would indicate that, far from largely an artistic element of the movie, paid product placement was the basis for a large majority of the time devoted to character smoking in Hollywood movies prior to 2000.

The MSA was followed by linear downward trends in the proportion of movies with any smoking and the number of character smoking scenes in movies with smoking. Here, it is less clear if the MSA was primarily responsible for these changes. While halting payments for movie product placement of cigarettes may have been responsible for setting them off, the downward trends extend for over a decade after the implementation of the MSA. It is equally likely that the ongoing public health campaign to limit movie smoking (see smokefreemovies.library.ucsf.edu) has substantially contributed to this continued decline, which extends well beyond the implementation of the MSA.^13^

In summary, an abrupt drop in movie brand placements and amount of screen time devoted to smoking depictions in Hollywood movies coincided with the implementation of externally enforced restrictions on paid cigarette product placement in movies by State Attorneys General. Given that such a large share of the smoking depicted may have been commercial in nature, the smoking scene (or any other scene with product placement elements) should be interpreted and regulated as if it is commercial, not artistic speech.

**What this paper adds**

- The Master Settlement Agreement (MSA) coincided with a sustained 77% reduction in average duration of character smoking in a scene (from 81 to 19 s), a change highly unlikely to be due to chance. It was also associated with significantly larger downward linear trends in the proportion of movies with smoking and counts of character smoking scenes.
- These correlational results suggest that elimination of paid product placement affected how movies were made vis-à-vis tobacco smoking by movie characters.

## References

[1] Centers for Disease Control and Prevention. Preventing tobacco use among youth and young adults: a report to the surgeon general. Atlanta, GA: Centers for Disease Control and Prevention, 2012. 22876391

[2] Glantz SA, Mitchell S, Titus K, et al Smoking in top-grossing movies—United States, 2010. MMWR Morb Mortal Wkly Rep 2011;60:909–13.

[3] Chapman S With youth smoking at historical lows, how influential is movie smoking on uptake? Addiction 2009;104:824–5; discussion 825–7 10.1111/j.1360-0443.2009.02568.x 19413794

[4] Snyder SL Movies and product placement: is Hollywood turning films into commercial speech? Univ Illinois Law Rev 1992;1:301–37.

[5] Master Settlement Agreement 1998 http://web.archive.org/web/20080625084126/http://www.naag.org/backpages/naag/tobacco/msa/msa-pdf/1109185724_1032468605_cigmsa.pdf (accessed 14 Oct 2015).

[6] Bergamini E, Demidenko E, Sargent JD Trends in tobacco and alcohol brand placements in popular US movies, 1996 through 2009. JAMA Pediatr 2013;167:634–9. 10.1001/jamapediatrics.2013.393 23712747 PMC3779902

[7] Adachi-Mejia AM, Dalton MA, Gibson JJ, et al Tobacco brand appearances in movies before and after the Master Settlement Agreement. JAMA 2005;293:2341–2. 10.1001/jama.293.19.2341 15900003 PMC1361304

[8] Jamieson PE, Romer D Trends in US movie tobacco portrayal since 1950: a historical analysis. Tob Control 2010;19:179–84. 10.1136/tc.2009.034736 20395408

[9] Berk RA Recent perspectives on the regression discontinuity designs. In: Bruinsma G, Weisburd D, eds Encyclopedia of criminology and criminal justice. New York: Springer, 2011:4335–50.

[10] Wood S https://cran.r-project.org/web/packages/mgcv/mgcv.pdf (accessed 14 Oct 2015).

[11] Mekemson C, Glantz SA How the tobacco industry built its relationship with Hollywood. Tob Control 2002;11 (Suppl 1):i81–91. 10.1136/tc.11.suppl_1.i81 11893818 PMC1766059

[12] Chapman S What should be done about smoking in movies? Tob Control 2008;17:363–7. 10.1136/tc.2008.027557 19029361

[13] Smokefreemovies 2015 http://smokefreemovies.ucsf.edu/sfm-ads (accessed 14 Oct 2015).

